# Comparison Between Urine Dipstick and Microscopic Examination Urinalysis With Urine Culture to Evaluate the Sensitivity and Specificity for Each in Diagnosing Urinary Tract Infection in Qassim Region, Saudi Arabia

**DOI:** 10.7759/cureus.59069

**Published:** 2024-04-26

**Authors:** Hana S Almuhanna, Ali M Alhojelan, Yasir A Al Rusayni, Muhannad A Almohanna, Hatem M AlDhalea, Abdullah A Aljulajil

**Affiliations:** 1 Emergency Medicine Department, Buraydah Central Hospital, Buraydah, SAU; 2 Emergency Medicine Department, King Fahad Specialist Hospital, Buraydah, SAU

**Keywords:** saudi arabia, emergency medicine, urine dipstick, urinalysis, urinary tract infection

## Abstract

Background: Urinalysis is the most popular test for evaluating emergency room patients with possible urinary tract infections (UTIs). Due to their speed and inexpensive cost, urine dipsticks are frequently performed in the Emergency Room. Although a urine dipstick test may be less expensive and time-saving than a laboratory study, it may not be accurate. The study evaluated the sensitivity and specificity of urine dipstick and microscopic urinalysis with a urine culture.

Methodology: A prospective, observational study was conducted on adults who visited the Emergency Department at King Fahd Specialist Hospital in Buraydah and reported having dysuria, urine urgency, or frequency, as well as suprapubic or costovertebral angle soreness. Patients who could not give a trustworthy history, had symptoms of vaginal discharge, or had taken antibiotics within the previous 72 hours were excluded.

Results: One hundred fifty-three urine samples were collected and examined using urinalysis and dipstick. In addition, 113 (73.86%) of 153 urine samples exhibited no growth in urine culture. With a count of nine, *Escherichia coli* (*E. coli*) was the most often isolated organism among the positive cultures (5.88%). *Klebsiella pneumoniae* was the second most common in our sample with eight (5.23%). The urine dipstick was shown to have an overall sensitivity of 0.79, specificity of 0.39, positive productive value (PPV) of 0.30, and negative productive value (NPV) of 0.85. Urinalysis exhibited a high sensitivity of 0.95 and a poor specificity of 0.21.

Conclusion: Our study showed that urine dipsticks may be more beneficial than urinalysis for ruling out urinary tract infections (UTIs), while urinalysis may be more helpful in verifying their presence.

## Introduction

Urinary tract infection (UTI) is the second most common infectious disease that affects about half of all individuals at some point [1]. An estimated 150 million people are affected by UTI annually, resulting in billions of dollars in economic costs worldwide [2]. UTI can manifest in various ways and is frequently diagnosed and treated in the Emergency Department (ED) [1]. Although there is no single definitive symptom that can diagnose a urinary tract infection (UTI), experiencing dysuria (painful urination), frequent urination, and urgency without vaginal discharge are all indicators that strongly suggest the presence of a UTI [1]. The common pathogens responsible for this significant public health issue include *Escherichia coli, Klebsiella pneumoniae, Proteus mirabilis, Enterococcus faecalis, Staphylococcus saprophyticus, Staphylococcus aureus, and Pseudomonas aeruginosa [2]*.

A urine examination, to be correlated with the history and physical exam, must be performed for a diagnosis. [3]. Urinalysis and urine dipstick tests are commonly used for testing, but interpreting the results can be challenging. Urine cultures should be taken for pregnant women with upper UTI [3]. The diagnosis of a UTI is confirmed with a urine analysis and culture, along with classic signs and symptoms of UTI. Urine cultures are not typically conducted in the ED, in any case. Results from urine cultures are not always immediately available [3]. While urine culture is considered the most reliable method for diagnosing UTI, at our centre, it is only available in the morning on weekdays. Consequently, many doctors now use urinalysis as the initial step in evaluating a UTI [3]. The most popular test for evaluating ED patients with possible UTI is urinalysis. However, this laboratory test can dramatically lengthen a patient’s stay in the ED. Although a urine dipstick test is more practical than a laboratory study, it should be equally or more accurate. When determining whether a person has a UTI, microscopic is a helpful supplement to a urine dipstick [3]. The urine dipstick is an accurate test that can be carried out with little training and is rapid and affordable. The results obtained can assist in determining whether to initiate empirical treatment or order a urine culture to confirm the diagnosis. The two most valuable urinalysis for diagnosing UTIs are leukocyte esterase and nitrite [3]. However, it is dubious to base therapy choices on urinalysis or urine dipsticks [4]. All patients with the presenting symptom of dysuria are frequently given an order for a urinalysis with manual dipstick and manual microscopy. However, many professionals in practice and certain hospitals only use microscopy when dipstick analysis reveals any abnormalities [3].

In this study, we will compare urine dipsticks and microscopic examination with urine culture to evaluate the sensitivity and specificity of each one of them to diagnose UTI in adults at King Fahad Specialist Hospital in Buraydah, Saudi Arabia.

## Materials and methods

The study was designed as a prospective, observational study to evaluate the accuracy of urine dipstick tests in predicting UTI in patients presenting to the Emergency Department (ED) at King Fahd Specialist Hospital in Buraydah, Al-Qassim. The sample size of 150 was determined by including all cases presented to the ED during the data collection period.

Patients who presented to the ED with symptoms of UTI, including dysuria, urine urgency, frequency, and suprapubic or costovertebral angle tenderness, patients who do not give an accurate history, and females with vaginal discharge, and those who take antibiotics within the previous 72 hours were excluded from the study. In our center, because the lab is available only in the morning and during workdays, the sample collection starts daily from 8 am to 4 pm.

The urine specimens collected at the time of the order were tested using reagent strips (ACON Biotech Hangzhou Co., Ltd, China) for leukocyte esterase, nitrite, glucose, bilirubin, and blood using dipstick tests. Additionally, a second aliquot of urine was sent to the hospital laboratory within one hour of collection for analysis. This analysis was conducted using LABUMAT (77 Elektronika Kft, Hungary), an automated urine chemistry analyzer, and UriSed 3 Pro (77 Elektronika Kft, Hungary), an automated urine sediment analyzer.

Furthermore, a urine culture specimen was obtained using sterile plastic containers of 1 mL volume. The collection method involved a midstream clean catch technique: the patient voided the initial portion of urine, collected the midstream urine specimen, and discarded the remaining portion.

Quantitative urine culture involves determining the colony count of the urine specimen by distributing 10 μl of urine onto a CLED-Agar (Watin-Biolife Factory, LOT: 204892) plate using standardized plastic loops. The plates were then incubated for 24 hours at 36 degrees Celsius. Colony counts are expressed as follows: one colony represents 10^2^ colony forming units per mL (cfu/mL), 10 colonies represent 10^3^ cfu/mL, 100 colonies represent 10^4^ cfu/mL, and 1000 colonies represent 10^5^ cfu/mL. Significant bacteriuria, defined as ≥ 10^5^ colony-forming units per mL in a midstream urine specimen, indicates a high likelihood of significant bacteriuria.

The data were collected in a Google Form by nurses and doctors, which included the results of the urine dipstick and the file number for each patient. Subsequently, after completing the data collection but before commencing the analysis, the authors obtained the results of urinalysis and urine culture using the file number associated with each sample in the initial form.

Dipstick and urinalysis data were compared with urine culture results to evaluate the accuracy of the tests in predicting UTIs. Sensitivity, specificity, and predictive values were calculated using various definitions of a positive for the combinations of tests mentioned above. Urine culture was used as the gold standard for comparison. Data were analyzed using statistical software (version 26) to calculate sensitivity, specificity, positive predictive value (PPV), and negative predictive value (NPV) for various combinations of leukocyte esterase, nitrite, glucose, bilirubin, and blood.

The comparative diagnostic value of test strips, conventional urinalysis and culture was evaluated in terms of sensitivity, specificity, PPV, and NPV, by using 2 x 2 tables, and urine culture as the gold standard. The PPV and NPV define the probability that the patient has or does not have a UTI. This study was approved by the General Directorate of Health Affairs Al-Qassem Region with an approval number 607/44/7262.

## Results

One hundred fifty-three urine samples were collected and examined using urinalysis and urine dipstick. Table 1 displays the frequency of abnormal urine dipstick and urinalysis results. Proteinuria was the most common aberrant finding on urine dipsticks in 56 samples (36.60%). Following this was hematuria, detected in 51 samples (33.33%). Glucosuria was detected in 31 samples (20.26%), whereas nitrite was detected in 15 (9.80%) samples. Leukocyte esterase was only present in five samples (3.27%). Proteinuria was the most common aberrant finding on urinalysis, occurring in 61 samples (39.87%). Hematuria was detected in 102 specimens (66.67%). Glucosuria was detected in 35 samples (22.88%), but nitrite was seen in just 10 (6.54%). Leukocyte esterase was present in 48 positive samples (31.37% ).

**Table 1:** Frequency of abnormal results on urine dipstick and urinalysis

Frequency of Abnormal Results on Urine Dipstick and Urinalysis				
Results	Urine dipstick		Urinalysis	
Variables	Frequency	Percent	Frequency	Percent
Normal	53	34.64%	26	16.99%
Protein	56	36.60%	61	39.87%
leukocyte esterase	5	3.27%	48	31.37%
nitrite	15	9.80%	10	6.54%
glucose	31	20.26%	35	22.88%
blood	51	33.33%	102	66.67%

**Figure 1:**
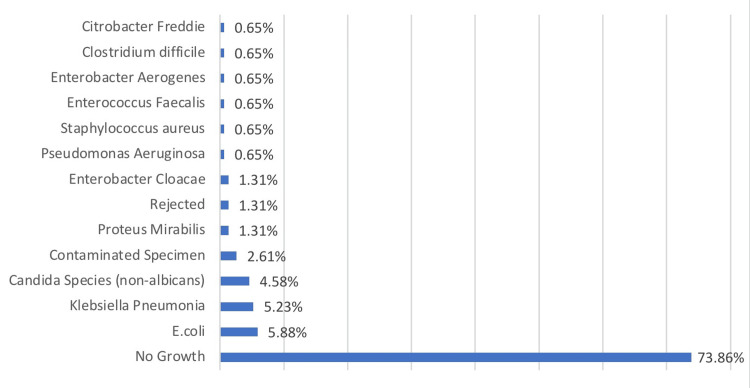
Results of urine culture

In addition, 113 (73.86%) of 153 urine samples exhibited no growth in urine culture. With a count of nine, *E.coli* was the most often isolated organism among the positive cultures (5.88%). *Klebsiella pneumoniae* followed this with eight (5.23%) and *Candida species (non-albicans)* with seven (4.58% ). *Proteus mirabilis (2),* *Enterobacter cloacae (2), Rejected *(insufficient quantity for proper processing)* (2), Pseudomonas aeruginosa (1), Staphylococcus aureus (1), Enterococcus fecalis (1), Enterobacter aerogenes (1), Clostridium difficile (1), and Citrobacter freundii (1) were the remaining positive cultures (1)* (Figure 1).

This study examined the sensitivity, specificity, PPV, and NPV of several urine dipstick tests. Table 2 summarizes the findings of the investigation. According to the findings, the accuracy of urine dipstick testing is dependent on the specific marker that is being measured. The leukocyte esterase + protein test, while having limited sensitivity and specificity, showed a high PPV and NPV. In contrast, only one patient tested positive for leukocyte esterase + nitrite, indicating that this test may be useful in confirming urinary tract infections in individuals with suspicious symptoms. The moderate sensitivity and specificity of the protein and blood suggest that they may help detect the presence of these indicators in urine samples. Nitrite and glucose had low sensitivity but reasonable specificity, indicating that they may be beneficial for excluding these indicators from urine samples. Significantly, the sensitivity and specificity of the tests differed considerably. The nitrite, for example, had a specificity of 0.93 but a sensitivity of only 0.18. The moderate sensitivity and specificity of the protein and blood suggest that they may help detect the presence of these indicators in urine samples. Nitrite and glucose had low sensitivity but reasonable specificity, indicating that they may be beneficial for excluding these indicators from urine samples. It is important to note that there was a notable difference in the sensitivity and specificity of the tests. The nitrite test, for example, had a specificity of 0.93 but a sensitivity of only 0.18. This demonstrates the significance of sensitivity and specificity when interpreting urine dipstick test findings. The urine dipstick was shown to have an overall sensitivity of 0.79, specificity of 0.39, PPV of 0.30, and NPV of 0.85.

**Table 2:** Sensitivity, specificity, PPV, and NPV of urine dipstick and its components compared to urine culture (PPV) positive predictive value, (NPV) negative predictive values.

Sensitivity, specificity, PPV, and NPV of urine dipstick and its components compared to urine culture				
Results	Sensitivity	Specificity	PPV	NPV
The test	0.79	0.39	0.30	0.85
Protein	0.66	0.73	0.45	0.87
Leukocyte esterase + Protein	0.13	0.01	1.00	1.00
Leukocyte esterase-nitrite (one case)	1	0	1	0
Nitrite	0.18	0.93	0.47	0.78
Glucose	0.18	0.79	0.23	0.75
Blood	0.50	0.72	0.37	0.81

This study compared the sensitivity, specificity, PPV, and NPV of urine tests to the results of culture analysis. Table 1 outlines the findings of the investigation. The results indicate that the accuracy of urinalysis tests varies depending on the marker being measured. Urinalysis exhibited a high sensitivity of 0.95 and a poor specificity of 0.21, suggesting that it may help verify the presence of urinary tract infections but may not be as successful as culture analysis in establishing their absence. Individual urine tests exhibited variable degrees of accuracy in comparison to culture analysis. The blood had a high sensitivity of 0.82 but a poor specificity of 0.38, indicating that it may be beneficial for detecting the presence of infection in urine samples but may not be as successful as culture analysis in ruling it out. Compared to culture analysis, the nitrite exhibited a high specificity of 1.00 and a high PPV of 1.00, indicating that it may be a valid marker for detecting the presence of infections in urine samples. Compared to culture analysis, the protein demonstrated moderate sensitivity and specificity, indicating that it may be effective for detecting the presence of infection in urine samples. The high sensitivity and specificity of the leukocyte esterase in comparison to culture analysis suggests that it may be effective for detecting the presence of infections in urine samples. Compared to culture analysis, the glucose demonstrated low sensitivity and specificity, indicating that it may not be a viable marker for detecting the presence of infection in urine samples. It is essential to note that the PPV and NPV of the individual tests differed significantly from the culture analysis. The PPV of the leukocyte esterase was only 0.10 when compared to culture analysis, whereas the PPV of the nitrite was 1.00 when compared to culture analysis. When interpreting urinalysis test results, it is essential to consider both sensitivity and specificity and the prevalence of the condition being evaluated.

**Table 3:** Sensitivity, specificity, PPV, and NPV of urinalysis and its components compared to urine culture (PPV) positive predictive value, (NPV) negative predictive values.

Sensitivity, specificity, PPV, and NPV of Urinalysis and its components compared to urine culture				
Results	Sensitivity	Specificity	PPV	NPV
Urinalysis in general	0.95	0.21	0.28	0.92
Protein	0.63	0.76	0.41	0.85
Leukocyte esterase	0.68	0.81	0.10	0.89
Leukocyte esterase + protein	0.42	0.91	0.62	0.83
Leukocyte esterase + nitrite	0.21	1.00	1.00	0.79
Nitrite	0.26	1.00	1.00	0.80
Glucose	0.16	0.75	0.17	0.72
Blood	0.82	0.38	0.30	0.86

## Discussion

Multiple factors make early identification of UTIs crucial. First, untreated UTIs can rapidly grow and cause complications like sepsis, especially for elderly people [5]. A bladder infection, for instance, might proceed to a kidney infection if not treated promptly, which can result in irreversible kidney damage and could be fatal [6,7]. Therefore, early detection and treatment of UTIs are crucial for preventing the spread of infection and minimizing the risk of consequences. Secondly, UTIs can give patients severe discomfort and anguish, which can be promptly alleviated by early detection and treatment [8]. UTI symptoms might include painful urination, frequent urination, and stomach pain, and can substantially impact the patient's quality of life [9]. Thirdly, early detection and treatment of UTIs can prevent the need for more intrusive and expensive treatments, including hospitalization and intravenous antibiotics [10]. If a UTI is diagnosed and treated promptly, it may be able to manage the infection with oral medicines and avoid more intrusive procedures. Finally, early detection and treatment of UTIs can aid in preventing the spread of infection, especially in healthcare settings [11].

Having a valuable and accurate technique for identifying UTIs in ED is crucial. As culture analysis can take several days to yield findings, there may be other viable methods for identifying urinary tract infections in ED settings [12]. Consequently, rapid and accurate diagnostic techniques with high sensitivity and specificity, such as urine dipsticks or urinalysis, can be beneficial in ED settings for detecting patients with suspected UTIs [13,14]. These tests can provide rapid answers and aid doctors in making prompt decisions on patient care and treatment. It is crucial to emphasize, however, that the accuracy of these tests should be evaluated in the context of the specific patient and their symptoms, and culture analysis may still be required in cases where there is a high suspicion of UTIs or if the initial tests are equivocal.

The findings of this study indicate that urine dipstick and urinalysis tests differ in their ability to detect UTIs compared to culture analysis. The sensitivity and specificity of urine dipsticks were determined to be 0.79 and 0.39, while those of urinalysis were 0.95 and 0.21. These results imply that urine dipsticks may be more beneficial than urinalysis for ruling out UTIs, while urinalysis may be more helpful in verifying their presence. These results are consistent with the results of some previous studies, which showed that the dipstick is more effective [15]. In a previous meta-analysis of 70 studies, the authors demonstrated that a negative urine dipstick test result for nitrites and leukocyte-esterase is sufficient to rule out infection in all populations [16]. Based on the results of urine dipstick tests, the leukocyte esterase + protein had a perfect PPV and NPV, indicating that it is highly accurate in confirming the presence or absence of urinary tract infections in individuals with suspicious symptoms. However, this test's sensitivity and specificity were limited; thus, it may not always detect the presence of a disease or may yield false-positive results. The moderate sensitivity and specificity of the protein and blood suggest that they help detect protein and blood in urine samples, which may be symptomatic of various diseases, including kidney disease or infection. The poor sensitivity but high specificity of the nitrite and glucose suggests that they may help exclude the presence of infection in urine samples, which can also be symptomatic of other diseases. This is similar to previous studies, which showed that the performance of nitrite alone showed a relatively low sensitivity of 27.7 (95% CI = 17.3-40.2) [15], and these results are similar to the results of many previous studies [17,18]. The poor sensitivity value for nitrite could be explained by not all isolates efficiently converting nitrate to nitrite or by the fact that patients may have voided urine before sample collection, resulting in a concentration of nitrites below the detection limit. Importantly, urine dipstick test results should be evaluated in the context of the patient's entire clinical presentation and may require confirmation through additional testing or clinical evaluation. The presence of blood tests in urinalysis showed good sensitivity but poor specificity, which is reported by previous studies [19]. The nitrite test's excellent specificity and positive predictive value suggest that it may be a valid marker for detecting the presence of infections in urine samples. Compared to culture analysis, the protein test revealed moderate sensitivity and specificity.

In contrast, the leukocyte esterase test demonstrated high sensitivity and specificity, indicating that they may help detect the presence of infections in urine samples [20,21]. In general, incorporating the outcomes of these examinations can help detect UTIs and support healthcare experts in making informed treatment decisions.

Study limitations

Our study has certain limitations because the data were only collected in one center and during the morning hours, which resulted in a limited sample size and the potential for bias.

## Conclusions

In conclusion, urine dipstick and urinalysis tests are frequently used in clinical practice to detect UTIs. However, the accuracy of these tests varies based on the type of test utilized, the prevalence of UTIs in the tested population, and the presence of confounding factors. When interpreting the results, the sensitivity and specificity of each test, the individual patient, and their symptoms must be addressed. Studies comparing the accuracy of urine dipstick and urinalysis tests for identifying UTIs indicate that, while these tests can provide doctors with useful information, they are not as reliable as culture analysis, which remains the gold standard for diagnosing UTIs. Therefore, the use of these tests should be examined in the context of the individual patient and their symptoms, and a culture study may be required in cases where there is a high suspicion of UTIs or where the results of the initial tests are inconclusive. Overall, the significance of early detection and treatment of UTIs cannot be emphasized since untreated UTIs can result in severe problems. Therefore, developing more reliable diagnostic techniques for detecting UTIs in ED settings is a crucial field of research with the potential to improve patient outcomes and reduce healthcare expenditures.
